# Incidence of Hyperlipidemia among Adults Initiating Antiretroviral Therapy in the HIV Outpatient Study (HOPS), USA, 2007–2021

**DOI:** 10.1155/2023/4423132

**Published:** 2023-11-30

**Authors:** Jun Li, Selom Agbobli-Nuwoaty, Frank J. Palella, Richard M. Novak, Ellen Tedaldi, Cynthia Mayer, Jonathan D. Mahnken, Qingjiang Hou, Kimberly Carlson, Angela M. Thompson-Paul, Marcus D. Durham, Kate Buchacz

**Affiliations:** ^1^Division of HIV Prevention, NCHHSTP, Centers for Disease Control and Prevention, Atlanta, GA, USA; ^2^Cerner Corporation, Kansas, MO, USA; ^3^Northwestern University Feinberg School of Medicine, Chicago, IL, USA; ^4^University of Illinois College of Medicine, Chicago, IL, USA; ^5^Lewis Katz School of Medicine at Temple University, Philadelphia, PA, USA; ^6^St. Joseph's Comprehensive Research Institute, Tampa, FL, USA; ^7^Division for Heart Disease and Stroke Prevention, NCCDPHP, Centers for Disease Control and Prevention, Atlanta, GA, USA

## Abstract

Current U.S. guidelines recommend integrase strand transfer inhibitor (INSTI)-based antiretroviral therapy (ART) as initial treatment for people with HIV (PWH). We assessed long-term effects of INSTI use on lipid profiles in routine HIV care. We analyzed medical record data from the HIV Outpatient Study's participants in care from 2007 to 2021. Hyperlipidemia was defined based on clinical diagnoses, treatments, and laboratory results. We calculated hyperlipidemia incidence rates and rate ratios (RRs) during initial ART and assessed predictors of incident hyperlipidemia by using Poisson regression. Among 349 eligible ART-naïve PWH, 168 were prescribed INSTI-based ART (36 raltegravir (RAL), 51 dolutegravir (DTG), and 81 INSTI-others (elvitegravir and bictegravir)) and 181 non-INSTI-based ART, including 68 protease inhibitor (PI)-based ART. During a median follow-up of 1.4 years, hyperlipidemia rates were 12.8, 22.3, 22.7, 17.4, and 12.6 per 100 person years for RAL-, DTG-, INSTI-others-, non-INSTI-PI-, and non-INSTI-non-PI-based ART, respectively. In multivariable analysis, compared with the RAL group, hyperlipidemia rates were higher in INSTI-others (RR = 2.25; 95% confidence interval (CI): 1.29–3.93) and non-INSTI-PI groups (RR = 1.89; CI: 1.12–3.19) but not statistically higher for the DTG (RR = 1.73; CI: 0.95–3.17) and non-INSTI-non-PI groups (RR = 1.55; CI: 0.92–2.62). Other factors independently associated with hyperlipidemia included older age, non-Hispanic White race/ethnicity, and ART without tenofovir disoproxil fumarate. PWH using RAL-based regimens had lower rates of incident hyperlipidemia than PWH receiving non-INSTI-PI-based ART but had similar rates as those receiving DTG-based ART, supporting federal recommendations for using DTG-based regimens as the initial therapy for ART-naïve PWH.

## 1. Introduction

People with HIV (PWH) experience high cardiovascular morbidity and mortality [1–3]. Hyperlipidemia, manifesting as high levels of low-density lipoprotein cholesterol (LDL-C), total cholesterol (TC), or triglycerides (TG), is a well-established risk factor for cardiovascular disease (CVD). Hyperlipidemia is common among PWH [2, 3]. In addition to the effects of HIV infection itself, smoking, physical inactivity, poor diet, aging, and use of specific antiretroviral therapies (ART) can increase the risk for hyperlipidemia [3, 4]. After its approval in 2007 by the Food and Drug Administration (FDA), raltegravir (RAL), the first integrase strand transfer inhibitor (INSTI), became widely used in the United States [5]. Subsequently, three other INSTIs—elvitegravir (EVG), dolutegravir (DTG), and bictegravir (BIC)—were approved in 2012, 2013, and 2018, respectively, and have been increasingly used because of their favorable efficacy and safety profiles [6–8]. In addition to the evolution in use of individual INSTIs, use of agents in other antiretroviral drug classes also shifted, with a notable transition from tenofovir disoproxil fumarate (TDF) to tenofovir alafenamide (TAF), with the former having marked effects on reducing serum lipids [9, 10]. INSTI-based regimens have been recommended as first-line therapy for PWH since 2015 [11]. However, it remains uncertain whether and which INSTI-based ART regimens yield more favorable lipid outcomes and how these compare to outcomes with non-INSTI-based ART in diverse populations of PWH in routine HIV care [12–15].

In 2003, the Infectious Disease Society of America recommended routine evaluation and management of hyperlipidemia in adults PWH receiving ART [3]. In 2015, the National Lipid Association released patient-centered hyperlipidemia treatment guidelines, which underscored the effect of HIV infection on CVD and recommended use of non-HDL-C (high-density lipoprotein cholesterol) (TC minus HDL-C, including LDL-C, intermediate-density and very LDL (VLDL), chylomicron remnants, and lipoprotein) as the primary indicator to stratify CVD risk categories [16, 17]. Our study aimed to (1) assess the effects of ART regimen use (RAL vs. other INSTIs and non-INSTIs) on hyperlipidemia among ART-naïve PWH and (2) determine ART regimen-related factors associated with incident hyperlipidemia, while adjusting for sociodemographic and clinical factors.

## 2. Methods

### 2.1. The HIV Outpatient Study

We analyzed data from the HIV Outpatient Study (HOPS), an ongoing, prospective cohort study that has been enrolling PWH (aged ≥ 18 years) since 1993. There were nine active HOPS sites in six U.S. cities during the time of this analysis (January 1, 2007−December 31, 2021): Chicago, IL (two sites); Denver, CO (three sites); Stony Brook, NY; Philadelphia, PA; Tampa, FL; and Washington, DC. The HOPS relies on routine medical record data generated during the course of routine HIV care. All HOPS data abstractors have healthcare-related backgrounds and have been trained in chart abstraction. After informed consent, information is abstracted from outpatient records at each visit, entered electronically, compiled centrally, and reviewed and edited before being analyzed. Abstracted data include sociodemographic characteristics, HIV transmission risk factors, diagnoses, HIV antiretroviral and other prescribed medications, laboratory values, hospitalizations, and deaths. The study protocol was reviewed and renewed annually by institutional review boards at participating sites and the Centers for Disease Control and Prevention.

### 2.2. Study Population

For this analysis, we used a HOPS dataset available as of June 30, 2022, and analyzed data through December 31, 2021, to allow for data entry lag. We limited inclusion to participants who were enrolled at any active HOPS sites in our study window (January 1, 2007–December 31, 2021), with at least two visits since HOPS enrollment and at least one visit within the study window. In addition, we required that the participants initiated highly active ART (a minimum of three drugs with at least one nucleoside reverse transcriptase inhibitor (NRTI)) during the study window, had complete ART history available within the HOPS database, and had at least two lipid measurements after ART initiation with LDL-C and HDL-C measured on the same day. We excluded the participants who received lipid lowering-treatment within six months prior to ART initiation and who had a charted diagnosis of “hypertriglyceridemia,” “hypercholesterolemia,” or “hyperlipidemia” at any point prior to ART initiation. Participants with abnormal laboratory measurements of TG (≥150 mg/dL), total cholesterol (≥200 mg/dL), non-HDL-C (≥160 mg/dL), or LDL-C (≥130 mg/dL) prior to ART were also excluded.

### 2.3. Definitions of Predictor and Outcome Variables

For this cohort analysis, the outcome variable was incident hyperlipidemia during the initial ART course, defined as the first instance during the follow-up of a clinical diagnosis including hypertriglyceridemia, hypercholesterolemia, or other lipid abnormalities; starting lipid-lowering treatment; or having elevated TG, TC, non-HDL-C, or LDL-C lab results. The initial ART regimen was characterized as INSTI-based ART (with RAL, DTG, or other INSTI agents (others)) and non-INSTI-based ART (protease inhibitors (PIs) and other agents (others)), respectively. The date of initial ART initiation was defined as the baseline date. The CD4+ T-lymphocyte count (CD4, cells/mm^3^), plasma HIV RNA levels (viral loads (VLs)), TC, TG, HDL-C, and LDL-C at the baseline were defined as the most recent lab results in the six months prior to or 30 days after their initial ART. Age in years at the baseline was calculated taking the difference of the date of ART initiation (baseline date) and date of birth.

### 2.4. Statistical Analysis

We described participants' sociodemographic, HIV-related, and lipid-related clinical characteristics at the baseline, unless otherwise specified. These characteristics included age, sex, race/ethnicity, HIV transmission risk group, insurance payer at the baseline, type of the HOPS site (public vs. private), nadir CD4, VL, years since HIV diagnosis, type of the first highly active ART regimen, alcohol use status, historical smoking status, family history of coronary heart disease (CHD), TC, TG, HDL-C, non-HDL-C, and LDL-C levels at the baseline, TDF and TAF use, and body mass index (BMI). We used Pearson's chi-square test to assess for differences in the frequencies for categorical variables and the nonparametric Kruskal–Wallis test to assess for differences in the distributions of continuous variables; we reported the median and range between quartiles 1 and 3 (*Q*1–*Q*3) for the continuous variables.

We calculated the incidence rate (IR) of hyperlipidemia by the ART regimen type (RAL-, DTG-, INSTI-others, non-INSTI-PI, and non-INSTI-non-PI) and other baseline characteristics. We conducted univariable Poisson regression to assess unadjusted associations of the hyperlipidemia incidence rate by the regimen type, age, sex, race/ethnicity (non-Hispanic White vs. others), type of the HOPS site, nadir CD4 count, BMI, and TDF use in the initial ART. In building the multivariable model, the ART regimen type along with all the aforementioned explanatory factors with *P* ≤ 0.2 in the univariable models was included in multivariable regression, but only those factors with *p* < 0.05 in this multivariable model were retained in the final model. In evaluating the Poisson regression model fit, we relied on Akaike information criterion (AIC) values. All analyses were conducted using SAS version 9.4 (SAS Institute, Cary, NC, USA). Results with *P* < 0.05 were considered statistically significant.

## 3. Results

### 3.1. Participant Selection

**Figure 1:**
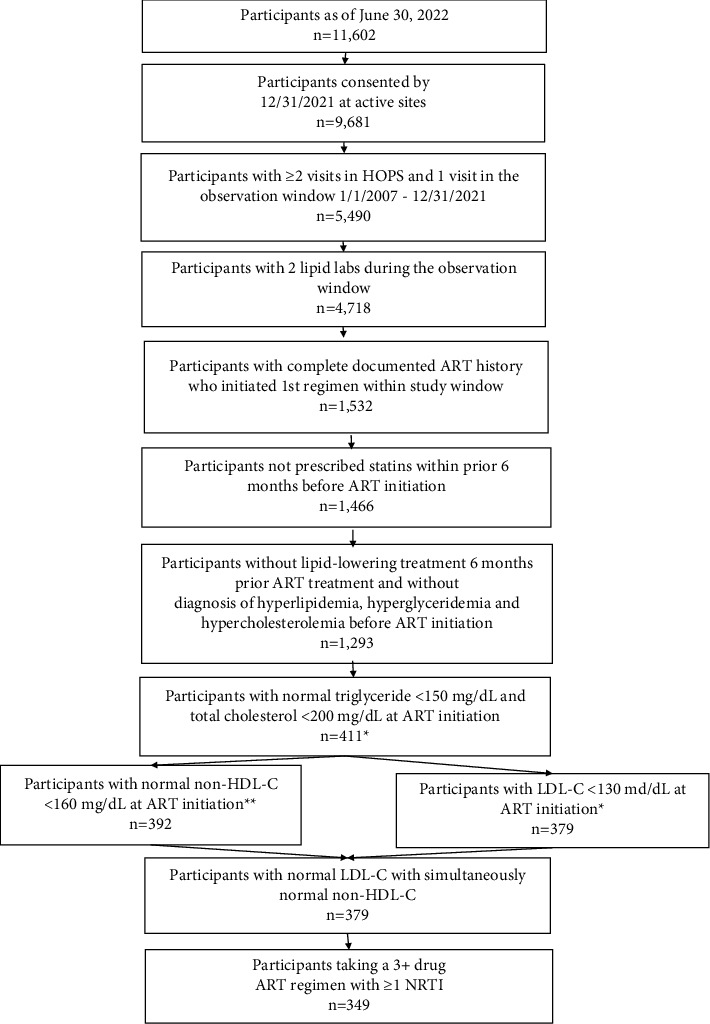
Selection steps of study participants from the HIV Outpatient Study, USA, 2007–2021. ^*∗*^LDL-C tests within a window of 6 months prior to ART initiation date and 30 days following the initiation were assessed. For the participants with multiple tests, the LDL-C value closest to the initiation date was used. ^*∗∗*^non-HDL-C was calculated as the total cholesterol result minus the HDL result. Total cholesterol and HDL results had to have been collected on the same day. Abbreviations: ART, antiretroviral therapy; HDL-C, high-density lipoprotein cholesterol; LDL-C, low-density lipoprotein cholesterol; NRTI, nucleoside reverse transcriptase inhibitor.

Among 11,602 PWH enrolled in the HOPS as of June 30, 2022, we selected 9,681 who were enrolled by December 31, 2021, among whom 5,490 had at least two visits and one visit in the observation window (January 1, 2007–December 31, 2021). We then identified 1,532 ART-naïve participants with at least 2 lipid measurements, a complete ART history, and who had initiated a first ART regimen with a minimum of three drugs within the study window. Of them, 1,293 participants did not receive lipid-lowering treatment within 6 months prior to ART initiation and did not have a diagnosis of hyperlipidemia, hypertriglyceridemia, or hypercholesterolemia before ART initiation. After excluding the participants with the TG level ≥150 mg/dL or TC level ≥200 mg/dL at the time of ART initiation, 411 participants remained. After further excluding 62 participants who had elevated non-HDL-C and LDL-C levels, there were 379 participants with normal levels of TG, TC, non-HDL-C, and LDL-C (non-HDL-C and LDL-C collected on the same day) at the time of ART initiation. Of them, 349 participants received at least three drug ART regimens including at least one NRTI (Figure 1).

### 3.2. Study Population Characteristics

In the resultant analysis population of 349 ARV-naïve participants without baseline hyperlipidemia, 168 were prescribed INSTI-based ART (36 RAL, 51 DTG, and 81 other INSTIs including 40 EVG and 41 BIC) and 181 non-INSTI-based ART (68 PIs and 113 other agents).

Table 1 describes demographic and clinical characteristics by the ART regimen type. In both groups (INSTI vs. non-INSTI), most participants were aged 18–44 years (80% vs. 75%), male (79% vs. 76%), not non-Hispanic-White (79% vs. 76%) persons, and gay, bisexual, and other men who have sex with men (collectively referred to as MSM) (58% vs. 55%). Median nadir CD4 was statistically higher for the INSTI group than for the non-INSTI group (354 vs. 289 cell/mm^3^, *P*=0.003). Comparatively, the median observed duration on the first regimen was statistically shorter for INSTI than for non-INSTI regimen types (1.1 years (*Q*1–*Q*3, 0.5, 2.3) vs. 1.6 years (*Q*1–*Q*3, 0.7, 3.4), *P*=0.025) (data not shown). In the INSTI group, all regimens included an INSTI and an NRTI. In the non-INSTI group, 37.6% received a regimen containing an NRTI and a PI and 62.4% received a regimen with an NRTI plus an NNRTI. During the first ART regimen, the INSTI and non-INSTI groups had 83 and 99 incident hyperlipidemia cases, with an incidence rate of 18.7 (*Q*1–*Q*3, 15.1, 23.2) and 14.3 (*Q*1–*Q*3, 11.8, 17.4) per 100 person years, respectively.

### 3.3. Correlates of Incident Hyperlipidemia

Table 2 depicts incidence rates and unadjusted and adjusted rate ratios of hyperlipidemia. During the observation, groups with RAL-, DTG-, INSTI-others-, non-INSTI-PI, and non-INSTI-non-PI-based ART had 22, 26, 35, 43, and 56 incident hyperlipidemia cases (incidence rates: 12.8, 22.3, 22.7, 17.4, and 12.6 per 100 person years, respectively). In unadjusted analysis, older age, non-Hispanic White race/ethnicity, public HOPS site, and not using TDF were positively associated with incident hyperlipidemia (all *P* < 0.05). In the multivariable analysis, compared with the RAL-based group, rates of hyperlipidemia were higher in the INSTI-others (RR = 2.25; 95% CI: 1.29–3.93) and non-INSTI-PI groups (RR = 1.89; CI: 1.12–3.19) but were not statistically higher in the DTG-based (RR = 1.73; CI: 0.95–3.17) and non-INSTI-non-PI-based groups (RR = 1.55; CI: 0.92–2.62). Other factors independently associated with hyperlipidemia included older age, non-Hispanic White race/ethnicity, and ART without TDF.

## 4. Discussion

Using 16 years of longitudinal data from a diverse population of HOPS participants in routine care, we found higher rates of incident hyperlipidemia among PWH receiving non-INSTI-PI- and INSTI-others (EVG and BIC)-based ART than RAL-based ART; however, no significant differences were observed for the DTG- and non-INSTI-non-PI groups compared with the RAL group.

Positive associations of hyperlipidemia with use of PI (including ritonavir)-based regimens have been reported in the literature [14, 18–20]. The effects on lipid outcomes also vary by specific agents used within antiretroviral classes; for example, lopinavir/ritonavir use was associated with a higher risk of dyslipidemia than atazanavir/ritonavir use [21]. The mechanism underlying this elevated risk might include excess fatty acid synthesis and hepatic steatosis caused by ritonavir [22]; protection of apolipoprotein B, an LDL component, from degradation by the proteasome [23]; and interactions with cellular retinoic acid binding protein 1 or lipoprotein receptor-related protein by PIs, leading to alterations in the lipid profile [15, 24].

Since RAL-based regimens were highly effective and well tolerated in both treatment-naïve and treatment-experienced patients including persons with multidrug-resistant HIV-1 infection [25, 29], the U.S. Department of Health and Human Services (DHHS) guidelines recommended the use of RAL in combination with tenofovir and emtricitabine as a preferred regimen for ART-naïve patients in 2009 [27]. With newer generations of FDA-approved INSTI agents, including DTG and BIC, in recent years, INSTIs have been incorporated into the majority of recommended first-line ART regimens for PWH. In 2020, due to its lower genetic barrier to resistance and higher pill burden than DTG- and BIC-containing ART regimens, RAL-based regimens were moved to the “recommended initial regimen in certain clinical situations” (BI) category [11]. Presently, DTG-based ART, including a 2-drug regimen (DTG/3TC), is recommended as a first-line regimen for most PWH [28].

Our study indicates that ART-naïve PWH in routine care with normal lipid profiles had lower rates of incident hyperlipidemia after initiating a RAL-based regimen than a PI-based regimen, a result which is supported by at least two PI to RAL switch studies [12, 29]. Based on the SWITCHMRK 1 and 2 randomized trials (lopinavir-ritonavir to RAL switch vs. not switch), Eron et al. reported that switching to RAL regimens was associated with significant reductions from the baseline in fasting TG, TC, and non-HDL-C compared with continuation of lopinavir-ritonavir-containing regimens at 12 weeks after the switch [29]. Similarly, Krikke et al. conducted an open-label cross over study and found that switching from Pl to RAL significantly decreased fasting TG, LDL-C, and TG levels at week 8 after the switch [12]. In addition, our study findings corroborate those from a recently published observational cohort study, RESPOND, which reported that ART-native PWH receiving INSTI had a lower incidence of dyslipidemia than those receiving boosted PIs [30]. In the primary analysis of the RESPOND study, dyslipidemia was defined as TC more than 240 mg/dl and/or HDL less than 35 mg/dl, TG more than 200 mg/dl, and/or initiation of lipid-lowering therapy. Although a higher incidence of dyslipidemia was observed among RAL recipients than those using DTG, the RESPOND study concluded that the effects of DTG and RAL on TC and TG were similar, which is consistent with our study findings and findings from the SPRING-2 study [30, 31]. The SPRING-2 study showed that DTG and RAL recipients exhibited similar lipid profiles and small increases in TC, LDL-C, and TG at 48 weeks of treatment for ART-naive PWH compared with baseline measurements [31]. However, recent studies suggest that use of DTG-based ART regimens is associated with greater weight gain than use of EVG-based regimens [32–34]. It is uncertain if such weight gain is also associated with lipid or other cardiometabolic disorders. Therefore, the effects of drugs in the INSTI class on lipid outcomes continue to represent an active and important area of clinical research. Lastly, we found that use of INSTI-other-based ART (EVG and BIC) was associated with a higher incidence of hyperlipidemia than RAL-based ART. Additional studies with a larger sample size and longer follow-up time are needed to confirm this finding.

As documented in earlier studies [9, 30, 35], TDF use has been associated with more favorable lipid outcomes compared to non-TDF-containing ART use, including TAF and abacavir (ABC). Studies have also shown that PWH taking TAF had a higher risk of hypercholesterolemia and hypertriglyceridemia than PWH taking TDF, and similarly, ABC use has been associated with a higher risk of hypercholesterolemia than TDF [9, 30].

This study is subject to at least three limitations. First, not all plasma lipid measurements were carried out in a fasting state, which may introduce some errors. Second, due to a limited sample size, we were not able to examine associations of hyperlipidemia with specific PI and NNRTI agents individually. We did assess the effect of DTG use on lipid outcomes and found no significant differences in incident hyperlipidemia compared with regimens of INSTI-others, non-INSTI-PI, and non-INSTI-non-PI, respectively. Because of a limited sample size, we could only evaluate and adjust for a few correlates in the multivariable analyses. Although, reassuringly, the INSTI and non-INSTI groups were broadly comparable by the baseline BMI, smoking, and alcohol history, we cannot rule out some residual confounding by unmeasured sociodemographic or other clinical or treatment history factors. Lastly, the rates and associations reported are for a select subset of ART-naïve patients without pre-existing hyperlipidemia, who were treated at nine urban HIV specialty clinics, and thus may not necessarily reflect the patterns for the broader PWH population in the United States.

The hyperlipidemic effects of use of some antiretroviral agents may, in part, contribute to cardiovascular risk among PWH [4, 36]. Initiating ART with more lipid-friendly ART regimens may provide an opportunity to prevent hyperlipidemia and possibly to reduce cardiovascular risk. Our central finding, i.e., the use of RAL-based regimens resulted in lower rates of incident hyperlipidemia than the use of non-INSTI-PI ART but similar rates as the use of DTG-based ART, supports federal recommendations for using DTG as an initial therapy for ART-naïve PWH.

## Figures and Tables

**Table 1:** Demographic and clinical characteristics by the ART regimen type among ART-naïve adults with HIV initiating ART in the HIV Outpatient Study, USA, 2007–2021 (*N* = 349).

Demographic and clinical characteristics	Total	Non-INSTI-based	INSTI-based	*P* value^1^
	*N*	*n* (%)	*n* (%)	
Total	349	181 (100)	168 (100)	
Age at baseline				0.30
18–44	270	136 (75.1)	134 (79.8)	
45+	79	45 (24.9)	34 (20.2)	
Age, years, median (*Q*1–*Q*3)	349	33.5 (25.6, 44.9)	31.8 (26.5, 41.9)	0.69
Sex at birth				0.52
Female	78	43 (23.8)	35 (20.8)	
Male	271	138 (76.2)	133 (79.2)	
Race/ethnicity				0.61
Non-Hispanic White	79	43 (23.8)	36 (21.4)	
Others	270	138 (76.2)	132 (78.6)	
HIV transmission risk group				0.73
MSM	196	100 (55.2)	96 (57.1)	
PWID	7	3 (1.7)	4 (2.4)	
Heterosexual	137	72 (39.8)	65 (38.7)	
Other/unknown	9	6 (3.3)	3 (1.8)	
Payer at baseline				0.019
Private	143	73 (40.3)	70 (41.6)	
Public	185	91 (50.3)	94 (56.0)	
No/other/unknown	21	17 (9.4)	4 (2.4)	
Type of the HOPS site				0.91
Public	103	54 (29.8)	49 (29.2)	
Private	246	127 (70.2)	119 (70.8)	
Nadir CD4 count at baseline (cells/mm^3^)				0.024
<200	112	65 (35.9)	47 (28.0)	
200–349	83	49 (27.1)	34 (20.2)	
350–499	77	38 (21.0)	39 (23.2)	
500+	70	25 (13.8)	45 (26.8)	
Unknown	7	4 (2.2)	3 (1.8)	
Median CD4 count (*Q*1–*Q*3)	312	289 (129.0, 417.0)	354 (180.0, 514.0)	0.003
HIV viral load at baseline (copies/mL)				0.75
<200	33	16 (8.8)	17 (10.1)	
200–999	24	12 (6.6)	12 (7.1)	
1,000–99,999	204	111 (61.3)	93 (55.4)	
≥100,000	77	38 (21.1)	39 (23.2)	
Unknown	11	4 (2.2)	7 (4.2)	
Median log_10_ viral load at baseline (*Q*1–*Q*3)	349	4.4 (3.7, 4.9)	4.5 (3.6, 5.0)	0.78
Years since HIV diagnosis to ART initiation, median (*Q*1–*Q*3)	349	0.3 (0.1, 1.2)	0.2 (0.1, 1.1)	0.003
Total cholesterol at baseline, median (*Q*1–*Q*3)	349	150.0 (128.0, 171.0)	145.5 (125.5, 167.5)	0.37
Total triglycerides at baseline, median (*Q*1–*Q*3)	349	92.0 (65.0, 116.0)	83 (62.5, 110.5)	0.17
HDL-C at baseline, median (*Q*1–*Q*3)	349	42.0 (34.0, 50.0)	42.0 (34.0, 50.0)	0.83
Non-HDL-C at baseline, median (*Q*1–*Q*3)	349	106.0 (86.0, 123.0)	100.0 (86.0, 120.5)	0.27
LDL-C at baseline, median (*Q*1–*Q*3)	349	87.0 (73.0, 107.0)	85.0 (69.5, 102.5)	0.58
Initial ART regimen				<0.0001
NRTI plus PI	68	68 (37.6)	0	
NRTI plus NNRTI	113	113 (62.4)	0	
NRTI plus INSTI	168	0	168 (100)	
TDF use				<0.001
Yes	230	163 (90.1)	67 (39.9)	
No (TAF and ABC)	119	18 (9.9)	101 (60.1)	
TAF use				<0.001
Yes	76	5 (2.8)	71 (42.3)	
No (TDF and ABC)	273	176 (97.2)	97 (57.7)	
Cigarette smoker at baseline				0.52
Ever	192	103 (56.9)	89 (53.0)	
Never	157	78 (43.1)	79 (47.0)	
Alcohol drinker at baseline				0.14
Current	86	51 (28.2)	35 (20.8)	
Not current	263	130 (71.8)	133 (79.2)	
Family history of CHD at HOPS enrollment				0.21
Yes	111	52 (28.7)	59 (35.1)	
No	238	129 (71.3)	109 (64.9)	
BMI at baseline				0.14
<25	180	95 (52.5)	85 (50.6)	
25–29.9	75	38 (21.0)	37 (22.0)	
30+	73	42 (23.2)	31 (18.5)	
Missing	21	6 (3.3)	15 (8.9)	
BMI, median (*Q*1–*Q*3)	349	24.5 (21.6, 29.3)	24.3 (21.6, 29.2)	0.92

*Note*. Baseline was defined as the date of ART initiation. ABC, abacavir; ART, antiretroviral therapy; BMI, body mass index; CHD, coronary heart disease; CI, confidence interval; HDL-C, high-density lipoprotein cholesterol; LDL-C: low-density cholesterol; INSTIs, integrase strand transfer inhibitors; *Q*1–*Q*3, range between quartiles 1 and 3; MSM, men who have sex with men; N/A, not applicable; NNRTI, nonnucleoside reverse transcriptase inhibitors; NRTI, nucleoside reverse transcriptase inhibitors; PI, protease inhibitor; PWIDs, people who inject drugs; TAF, tenofovir alafenamide; TDF, tenofovir disoproxil fumarate. ^1^Chi-square or Fishers exact test for categorical variables and the Wilcoxon two-sample test for continuous variables.

**Table 2:** Hyperlipidemia^1^ incidence rates (per 100 person years) and rate ratios among ART-naïve adults with HIV initiating ART in the HIV Outpatient Study, USA, 2007–2021 (*N* = 349).

	IR (95% CI)	RR (95% CI)	*P* value^2^	aRR^3^ (95% CI)	*P* value^2^
Total	16.0 (13.9, 18.6)				
ART regimen type					
RAL	12.8 (8.4–19.4)	Ref		Ref	
DTG	22.3 (15.2–32.7)	1.75 (0.99, 3.08)	0.05	1.73 (0.95, 3.17)	0.07
INSTI-others^4^	22.7 (16.3–31.7)	1.78 (1.04, 3.04)	0.03	2.25 (1.29, 3.93)	0.005
Non-INSTI-PI	17.4 (12.9–23.4)	1.36 (0.81, 2.27)	0.24	1.89 (1.12, 3.19)	0.018
Non-INSTI-non-PI	12.6 (9.7–16.4)	0.99 (0.60, 1.62)	0.96	1.55 (0.92, 2.62)	0.09
Age/10 years	NA	1.31 (1.02, 1.64)	<0.0001	1.38 (1.22, 1.57)	<0.0001
BMI/5 units	NA	1.11 (0.89, 1.38)	0.06		
Race/ethnicity					
Non-Hispanic White	25.6 (19.7, 33.4)	1.86 (1.35, 2.55)	<0.0001	2.22 (1.61, 3.08)	<0.0001
Others	13.8 (11.6, 16.4)	Ref		Ref	
Type of HOPS site					
Public	24.5 (19.2, 31.1)	Ref			
Private	13.4 (11.2, 16.1)	0.55 (0.41, 0.74)	<0.0001		
Sex at birth					
Female	15.7 (11.8, 21.0)	0.97 (0.69, 1.36)	0.86		
Male	16.2 (13.7, 19.1)	Ref			
Nadir CD4 count/100 cells	NA^3^	0.95 (0.89, 1.03)	0.23		
TDF use					
Yes	13.2 (11.0, 15.8)	Ref		Ref	
No (TAF and ABC)	27.1 (21.2, 34.7)	2.05 (1.51, 2.79)	0.001	1.84 (1.23, 2.75)	0.003

ABC, abacavir; ART, antiretroviral therapy; aRR, adjusted rate ratios; BMI, body mass index; CI, confidence interval; DTG, dolutegravir; RAL, raltegravir; INSTIs, integrase strand transfer inhibitors; IR, incidence rate; NA, not applicable; PI, protease inhibitor; RR, rate ratio; TAF, tenofovir alafenamide; TDF, tenofovir disoproxil fumarate. ^1^Hyperlipidemia is defined based on diagnosis, starting antilipidemic treatments, non-HDL-C ≥160 mg/dL, LDL-C ≥130 mg/dL, triglycerides ≥150 mg/dL, or total cholesterol ≥200 mg/dL. ^2^*P* values were derived from the chi-squared test. ^3^aRR was derived from Poisson regression, and the model was adjusted for age/10 years, race/ethnicity, type of the HOPS site, and TDF use. The ^4^INSTI-others group included bictegravir and elvitegravir.

## Data Availability

The data used to support the findings of this study are not freely available due to patient privacy.

## Abbreviations

ART: Antiretroviral therapy
HDL-C: High-density lipoprotein cholesterol
LDL-C: Low-density lipoprotein cholesterol
NRTI: Nucleoside reverse transcriptase inhibitors.
